# Commentary: Sodium Glucose Cotransporter 2 Inhibitors Reduce the Risk of Heart Failure Hospitalization in Patients With Type 2 Diabetes Mellitus: A Systematic Review and Meta-Analysis of Randomized Controlled Trials

**DOI:** 10.3389/fendo.2021.664502

**Published:** 2021-03-26

**Authors:** Mei Qiu, Liang-Liang Ding, Ze-Lin Zhan, Hai-Rong Zhou

**Affiliations:** ^1^Department of General Medicine, Shenzhen Longhua District Central Hospital, Shenzhen, China; ^2^Department of Endocrinology, First Affiliated Hospital of Yangtze University, Jingzhou, China; ^3^Class 3, Clinical Medicine, Grade 2019, The Second Clinical Medical College, Southern Medical University, Guangzhou, China

**Keywords:** SGLT2 inhibitors (gliflozins), type 2 diabetes (clinical domain), heart failure - pharmacological treatment - systolic dysfunction, cardiovascular death, all-cause death

## Introduction

We read with interest a meta-analysis (1) recently published in “Frontiers in Endocrinology” conducted by Zhang et al. In this study (1), Zhang and colleagues included eight randomized controlled trials (RCTs) comparing sodium-glucose cotransporter 2 inhibitors (SGLT2is) with placebo in patients with type 2 diabetes (T2D), and performed a meta-analysis to produce a pooled risk ratio (RR) and 95% confidence interval (CI) of SGLT2is versus placebo in reducing four cardiovascular endpoints.

The authors in this study (1) concluded that SGLT2is would be an ideal choice for T2D patients with heart failure (HF) because they found that SGLT2is significantly reduced hospitalization for heart failure (HHF), major adverse cardiovascular events (MACE, a composite of cardiovascular death, myocardial infarction, or stroke), and cardiovascular death (CVD) versus placebo in T2D patients. On the other hand, they concluded that SGLT2is did not significantly affect all-cause death (ACD) because they produced the nonsignificant 95% CI of RR (SGLT2is versus placebo: RR 0.77, 95% CI 0.59-1.01) for ACD. In my opinion, these two conclusions are not rigorous. First, they cannot conclude that SGLT2is are an ideal choice for T2D patients with HF until they assess the efficacy of SGLT2is in this T2D subgroup of concomitant HF and identify the obvious effectiveness. Second, using RR as drug effect is not accurate enough; all the original studies included in the meta-analysis (1) used hazard ratio (HR) as drug effect and RR only contains the status of the occurrence of events, but fails to contain the time when events happen. HR, on the other hand, contains both.

Thus, to validate and further extend the findings in the meta-analysis by Zhang et al. (1), we implemented this further quantitative synthesis study by carrying out a meta-analysis stratified by the status of HF based on the data of HRs and 95% CIs as reported in the original studies. Moreover, we additionally incorporated the recently published SOLOIST-WHF trial (2), in addition to the eight RCTs included in the study by Zhang et al. (1), because this trial (2) contributed to the relevant data of the T2D subgroup of concomitant HF.

## Findings Derived From Our Meta-Analysis

**Figure 1:**
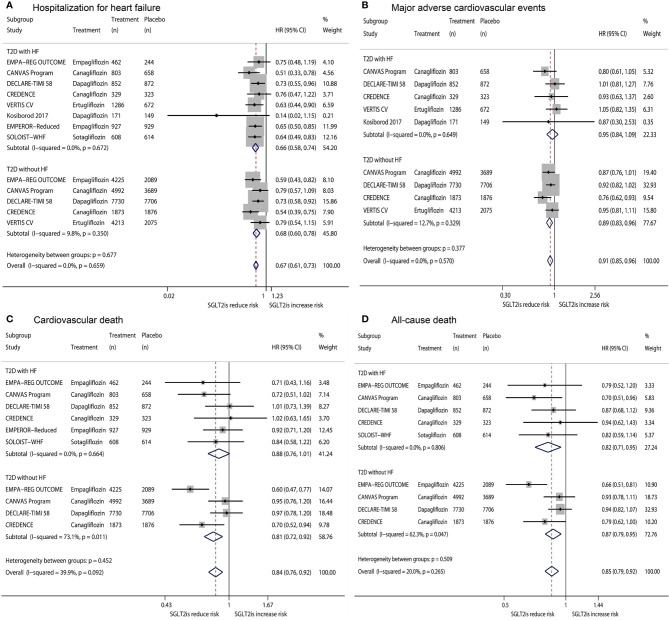
Fixed-effects meta-analysis of the effects of SGLT2is on hospitalization for heart failure **(A)**, major adverse cardiovascular events **(B)**, cardiovascular death **(C)**, and all-cause death **(D)** in T2D patients, stratified by the status of HF. SGLT2is = sodium-glucose cotransporter 2 inhibitors. T2D, type 2 diabetes; HF, heart failure; HR, hazard ratio; CI, confidence interval.

**Figure 1** shows the results of fixed-effects meta-analysis of the effects of SGLT2is on HHF, MACE, CVD, and ACD in T2D patients, stratified by the status of HF. Compared with placebo, SGLT2is significantly reduced HHF in T2D patients with HF (HR 0.66, 95% CI 0.58-0.74, P <0.001) and in T2D patients without HF (HR 0.68, 95% CI 0.60-0.78, P <0.001), with the nonsignificant subgroup effect (P_subgroup_ =0.677) (**Figure 1A**). SGLT2is did not significantly affect MACE in T2D patients with HF (HR 0.95, 95% CI 0.84-1.09, P =0.492) but significantly reduced MACE in T2D patients without HF (HR 0.89, 95% CI 0.83-0.96, P =0.002), with the nonsignificant subgroup effect (P_subgroup_ =0.377) (**Figure 1B**). SGLT2is produced a reduced trend in the risk of CVD in T2D patients with HF (HR 0.88, 95% CI 0.76-1.01, P =0.070) and significantly reduced CVD in T2D patients without HF (HR 0.81, 95% CI 0.72-0.92, P =0.001), with the nonsignificant subgroup effect (P_subgroup_ =0.452) (**Figure 1C**). SGLT2is significantly reduced ACD in T2D patients with HF (HR 0.82, 95% CI 0.71-0.95, P =0.007) and in T2D patients without HF (HR 0.87, 95% CI 0.79-0.95, P =0.002), with the nonsignificant subgroup effect (P_subgroup_ =0.509) (**Figure 1D**).

Due to substantial heterogeneity observed in fixed-effects meta-analyses of CVD and ACD, an additional meta-analysis using a random-effects model was conducted for the two outcomes to assess the robustness of pooled analysis results. **Figure S1** (random-effects model for CVD) shows that SGLT2is significantly reduced CVD (Overall HR 0.83, 95% CI 0.74-0.94, P <0.001) in T2D patients regardless of whether they were with/without HF (P_subgroup_ =0.452). **Figure S2** (random-effects model for ACD) shows that SGLT2is significantly reduced ACD (Overall HR 0.84, 95% CI 0.77-0.92, P <0.001) in T2D patients regardless of whether they were with/without HF (P_subgroup_ =0.509). The results revealed by random-effects model were consistent with those shown by the fixed-effects model. All the data extracted from included studies and analyzed in the present meta-analysis are given in **Supplementary Material 1**.

## Discussion

Based on the HRs and 95% CIs derived from nine RCTs, consisting of eight RCTs included in the meta-analysis by Zhang et al. (1) and the SOLOIST-WHF trial (2), we conducted a further meta-analysis to evaluate the efficacy of SGLT2is on four cardiovascular outcomes (HHF, MACE, CVD, and ACD) in the two T2D subgroups of T2D patients with HF and T2D patients without HF. Accordingly, we identified that SGLT2is versus placebo significantly reduced HHF (HR 0.66, 95% CI 0.58-0.74) and ACD (HR 0.82, 95% CI 0.71-0.95) and showed a decreased trend in the risk of CVD (HR 0.88, 95% CI 0.76-1.01) but did not significantly affect MACE (HR 0.95, 95% CI 0.84-1.09) in T2D patients with HF, while SGLT2is significantly reduced the four endpoints in T2D patients without HF. These findings support that SGLT2is should be used in T2D patients with HF as well as in T2D patients without HF to prevent the occurrence of these mortality and cardiovascular outcomes.

Two prior meta-analyses (3, 4), including three to five RCTs revealed that SGLT2is versus placebo significantly reduced the composite outcome of CVD or HHF in T2D patients regardless of whether they were with/without HF, but failed to assess the two individual outcomes according to the status of HF. Our present meta-analysis, including nine RCTs, further demonstrates the efficacy of SGLT2is on three individual outcomes (i.e., HHF, CVD, and ACD) in T2D patients independent of the status of HF.

Moreover, a meta-analysis (4) from our research team also confirmed that SGLT2is significantly reduced HF and renal failure composite outcomes in T2D patients regardless of whether they were with/without HF and regardless of whether they were had chronic kidney disease (CKD). A meta-analysis (5) based on the two trials of DAPA-HF (6) and EMPEROR-Reduced (7) conducted in HF patients identified the effectiveness of SGLT2is in reducing HF composite outcome among HF patients independent of T2D and CKD status. The DAPA-CKD trial (8) revealed that dapagliflozin produced similar benefits on the renal and cardiovascular composite endpoint for CKD patients regardless of T2D status. According to the above findings from previous studies (4–8), SGLT2is should be recommended in T2D patients with/without CKD, in HF patients with/without T2D/CKD, and in CKD patients with/without T2D to prevent cardiovascular, renal, and mortality events.

In the present meta-analysis, we conducted subgroup analyses stratified by the presence of HF or not, but failed to carry out more specific subgroup analyses stratified by HF with reduced ejection fraction (HFrEF), HF with mid-range ejection fraction (HFmrEF), or HF with preserved ejection fraction (HFpEF). Further studies performing these analyses would be clinically meaningful.

The findings revealed by the present meta-analysis suggest that SGLT2is should be used in T2D patients with/without HF, while those revealed by previous meta-analyses and large randomized trials suggest that SGLT2is should be also recommended in T2D patients with/without CKD, in HF patients with/without T2D/CKD, and in CKD patients with/without T2D to prevent cardiovascular, renal, and mortality events.

## Author Contributions

Conceptualization: MQ. Data Collection: MQ, L-LD, and H-RZ. Formal Analysis: L-LD and H-RZ. Validation: MQ, and Z-LZ. Writing – Original Draft Preparation: MQ. Writing – Review and Editing: H-RZ and L-LD. All authors contributed to the article and approved the submitted version.

## Funding

This work is supported by the Shenzhen Key Medical Discipline Construction Fund (SZXK063).

## Conflict of Interest

The authors declare that the research was conducted in the absence of any commercial or financial relationships that could be construed as a potential conflict of interest.

## Supplementary Material

Supplementary Figure 1 Random-effects meta-analysis of the effect of SGLT2is on cardiovascular death in T2D patients, stratified by the status of HF.

Supplementary Figure 2 Random-effects meta-analysis of the effect of SGLT2is on all-cause death in T2D patients, stratified by the status of HF.

Supplementary Material 1 Data extracted from included studies.
