# Supplementary figures and images for: Commentary: Sodium Glucose Cotransporter 2 Inhibitors Reduce the Risk of Heart Failure Hospitalization in Patients With Type 2 Diabetes Mellitus: A Systematic Review and Meta-Analysis of Randomized Controlled Trials
Source: Front Endocrinol (Lausanne). 2021 Mar 26;12:664502. doi: 10.3389/fendo.2021.664502 (PMC8033164; doi:10.3389/fendo.2021.664502)

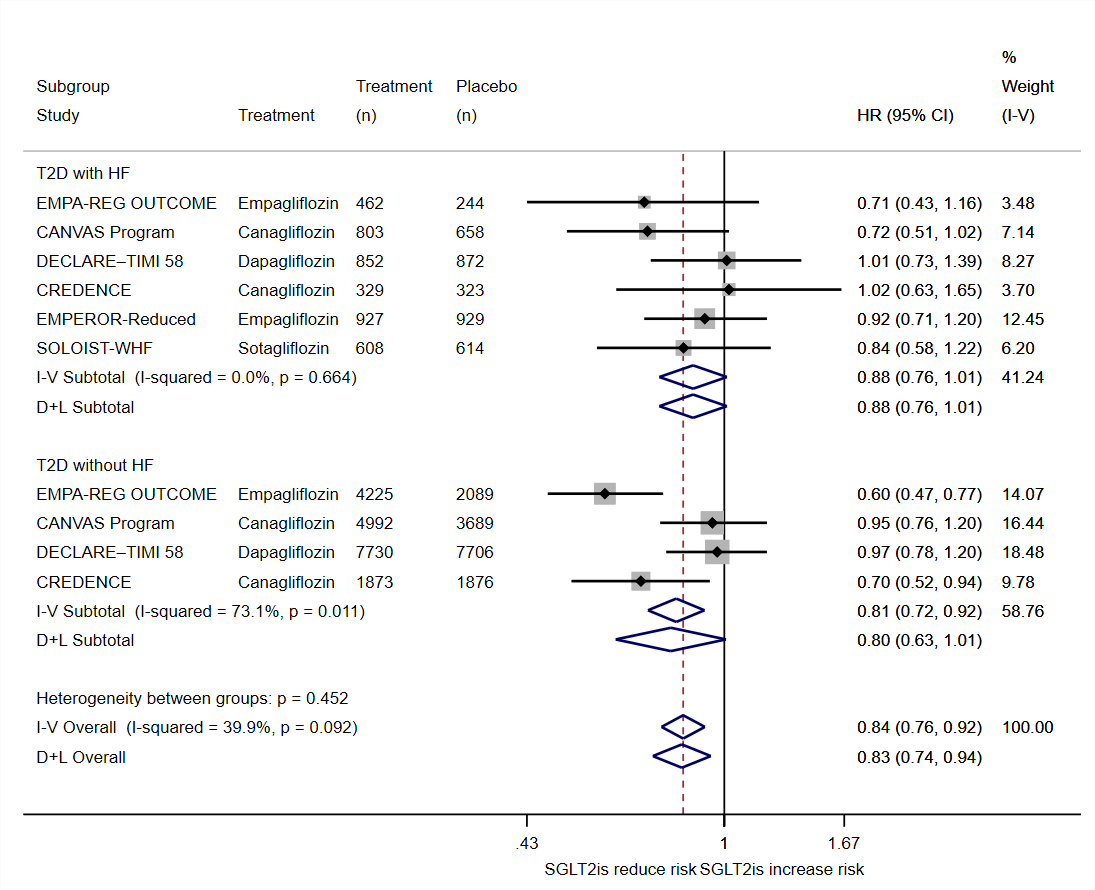

Supplement: Supplementary Figure 1 — Random-effects meta-analysis of the effect of SGLT2is on cardiovascular death in T2D patients, stratified by the status of HF. [file Image_1.tif]

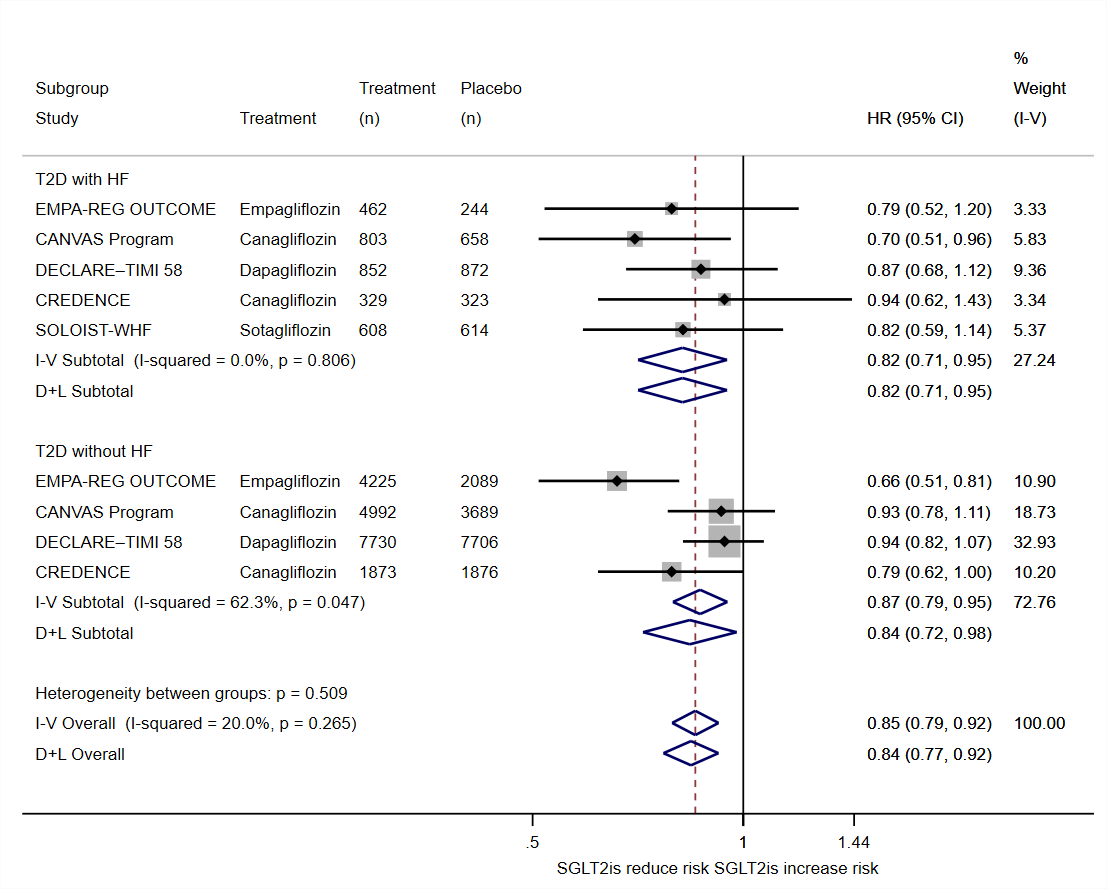

Supplement: Supplementary Figure 2 — Random-effects meta-analysis of the effect of SGLT2is on all-cause death in T2D patients, stratified by the status of HF. [file Image_2.tif]
